# Large language model-driven time-series forecasting of financial network indicators

**DOI:** 10.3389/frai.2026.1722121

**Published:** 2026-01-29

**Authors:** Mini Han Wang, Ying Yeung

**Affiliations:** 1 The Chinese University of Hong Kong, Hong Kong, Hong Kong SAR, China; 2 Frontiers Science Computing Center, Zhuhai Institute of Advanced Technology Chinese Academy of Sciences, Zhuhai, Guangdong, China; 3 School of Economic, Shenzhen Polytechnic University, Shenzhen, Guangdong, China

**Keywords:** degree centralization, financial network analysis, large language models (LLMs), residual density, retrieval-augmented generation (RAG), stock information network, systemic risk and market stability, time-series forecasting

## Abstract

**Introduction:**

Financial markets operate as dynamic networks in which institutional cross-holdings shape the diffusion of information and the propagation of risk. Forecasting the evolution of stock information networks is critical for anticipating herding behavior and safeguarding systemic stability, yet remains challenging due to high-dimensional heterogeneity, structural non-stationarity, and the need for economically interpretable predictions.

**Methods:**

Using a quarterly fund–stock holding panel from 2016 to 2024, we construct time-indexed bipartite fund–stock graphs and project them onto the stock layer. From these graphs, we compute two key network indicators: degree centralization (cen_d), capturing market-wide concentration, and residual density (den), reflecting firm-level anomalies. We then develop a large language model (LLM)–enhanced forecasting framework that transforms numeric time series and textual fund disclosures into promptable sequences, incorporates retrieval-augmented historical context, and performs multi-step forecasting of both cen_d and abnormal den spikes.

**Results:**

Extensive experiments show that the proposed LLM-based framework significantly reduces mean absolute error and root mean square error, and improves directional accuracy, compared with ARIMA, Prophet, and Temporal Fusion Transformer benchmarks. Attention-weight analysis further indicates that the model assigns higher importance to historical quarters characterized by sharp fund co-movement or policy shocks.

**Discussion:**

These findings demonstrate that LLM-driven time-series forecasting can provide early warnings of systemic risk and generate economically interpretable insights for investors and regulators. The results highlight the broader potential of language-informed graph forecasting as a new paradigm for financial market surveillance and policy design.

## 1 Introduction

Financial markets function as complex, interdependent systems in which information (Maurya et al., 2025), capital, and risk propagate through evolving networks of institutional investment. When multiple funds hold overlapping portfolios, the resulting stock information network (Zhai et al., 2025) creates structural linkages among equities that channel information (Chen et al., 2022) and magnify shocks (Wang et al., 2020). Sudden changes in network topology, such as abrupt increases in degree centralization (Zhai et al., 2025), can precipitate herding behavior, reduce market resilience, and heighten systemic risk. Understanding and forecasting the evolution of such networks is therefore a central challenge for both investors (Xue et al., 2021) and regulators seeking to safeguard market stability.

Parallel to these developments, time-series forecasting has been transformed by large language models (LLMs) (Chen et al., 2025). Originally designed for natural language tasks, LLMs are now recognized for their ability to capture long-range dependencies (Wang, 2025a), integrate heterogeneous data types, and generate coherent, structured predictions (Xiao et al., 2025). Their natural capacity to reason over both numbers and text makes them ideal for modeling the intricate combination of numerical indicators and qualitative market narratives that drive the dynamics of financial networks. Despite this promise, applications of LLMs to the forecasting of stock information networks remain limited, leaving significant methodological opportunities unexplored.

The present study tackles a set of interrelated challenges that have constrained previous attempts to forecast the evolution of stock information networks (Huang et al., 2025). These challenges arise from the structural complexity of fund–stock relations, the dynamic nature of financial markets, and the imperative for interpretability in high-stakes economic applications (Bao et al., 2025). A first obstacle concerns high-dimensional and heterogeneous data (Wang et al., 2022). The relationships between institutional funds and the stocks they hold embody a mixture of structured quantitative information, such as quarterly holding ratios, transaction volumes, and market capitalization, and unstructured qualitative signals, including fund strategies, portfolio narratives, and company-specific announcements (Liu, 2024). Capturing predictive dependencies across such heterogeneous data types requires models capable of fusing numeric time series with textual descriptions, a capacity that is only partially addressed by conventional econometric and machine-learning tools (Nguyen et al., 2025). A second difficulty is the pronounced structural non-stationarity of market networks. Stock–fund linkages can reorganize abruptly under the influence of policy reforms, macroeconomic shocks, or large-scale reallocation of institutional capital. These sudden regime shifts generate nonlinear patterns and long-range dependencies (Afzal et al., 2025) that violate the assumptions of classical linear time-series models and challenge the stability of even sophisticated deep-learning predictors. Accurate forecasting therefore demands methods that can flexibly adapt to evolving network topologies and capture rare but consequential structural breaks. A third challenge lies in ensuring interpretability and economic meaning. Forecasts must do more than produce accurate numerical trajectories; they must also illuminate the mechanisms of information diffusion, herding behavior, and systemic risk propagation so that investors, regulators, and policy makers can translate model outputs into effective preventive or corrective action. Black-box (Wang et al., 2023a) predictions without clear economic rationale risk undermining trust and limiting practical adoption.

Existing forecasting approaches address these issues only partially. Classical econometric models such as Autoregressive Integrated Moving Average (ARIMA) and conventional deep-learning architectures (Liu, 2025) like LSTM (Ricchiuti and Sperli, 2025) or standard transformers can model certain temporal patterns, yet they remain limited in their ability to jointly process multi-source numerical and textual sequences while providing transparent, economically interpretable explanations. Overcoming these limitations motivates the present study’s integration of graph-based (Bai et al., 2023) financial metrics with the representational and reasoning power of large language models (Zhao et al., 2025).

To overcome these limitations, we construct a quarterly fund–stock holding panel spanning 2016–2024 and use it to build time-indexed bipartite networks that are projected onto the stock layer. From each network we derive two pivotal indicators: degree centralization (cen_d) (Zhang et al., 2025), which measures the concentration of network connections and signals market-wide herding, and residual density (den), which isolates stock-level anomalies in cross-holding intensity after adjusting for firm size (Huang et al., 2025). Anticipating fluctuations in these indicators enables early warning of market instability and provides a scientific basis for proactive risk management.

Building on these data, we propose a large language model–enhanced forecasting framework that converts both numeric time series and textual fund information into structured prompts, enriches them with retrieved historical analogs, and predicts multi-step future values using GPT-style transformers. By directly comparing this framework with leading benchmarks, ARIMA, Prophet, and the Temporal Fusion Transformer (TFT) (Thundiyil and Picone, 2025), we show that LLMscan materially improve forecasting accuracy and reveal the economic mechanisms underlying network changes (Xiao et al., 2025).

Empirical results demonstrate that the LLM-based model achieves lower mean absolute and root mean square errors and higher directional accuracy in predicting next-quarter degree centralization than all baseline approaches. At the individual-stock level, the method detects abnormal residual-density spikes with superior precision and recall, offering a reliable early-warning mechanism for idiosyncratic risk and cross-holding contagion. Attention-weight analysis further reveals that the LLM naturally highlights historical periods of heightened fund co-movement or policy-driven capital shifts, providing transparent evidence of how market narratives shape network evolution.

These findings carry far-reaching implications for financial practice and regulation. Accurate forecasts of rising degree centralization provide investors with actionable insights to rebalance portfolios before herding-induced volatility materializes (Glickman and Sharot, 2025). Reliable detection of abnormal residual densities identifies stocks that may act as critical conduits for systemic risk, enabling regulators to strengthen market surveillance and refine stress-testing protocols. Unlike existing financial forecasting approaches that rely solely on numerical signals or treat textual information as auxiliary sentiment features, this study introduces a language-model–driven forecasting paradigm that explicitly reasons over network structure, temporal dynamics, and historical narratives. By integrating graph-derived indicators with retrieval-augmented textual context, the proposed framework captures both gradual structural evolution and abrupt regime shifts—such as policy shocks and fund co-movement events—that are difficult to detect using conventional econometric or deep learning models. Importantly, we demonstrate that these gains arise not from the transformer architecture alone, but from LLM-style prompting, contextual retrieval, and attention-driven temporal reasoning. It shows how language-informed graph forecasting can advance the transparency and explanatory power of artificial intelligence (Bao et al., 2024) in economics and finance.

The remainder of this article is organized as follows. Section 2 (Data and Preprocessing) introduces the quarterly fund–stock holding panel that underpins the study, explains how bipartite fund–stock networks are constructed and projected onto the stock layer, and defines the key structural indicators, degree centralization (cen_d) and residual density (den). Section 3 (Methodology) presents the LLM–enhanced forecasting framework, detailing prompt construction, retrieval-augmented generation, and the benchmark models used for rigorous comparison. Section 4 (Experiments) describes the design of the empirical evaluation, including next-quarter cen_d prediction and abnormal den spike detection, together with the rolling-origin protocol and accuracy metrics. Section 5 (Results and Discussion) reports the forecasting gains of the LLM model, interprets attention weights to uncover the market mechanisms driving network evolution, and explores implications for systemic risk and information diffusion. Section 6 (Conclusions) summarizes the main contributions, highlights financial and regulatory significance, and identifies avenues for future research while acknowledging the study’s limitations.

## 2 Materials and methods

To forecast the evolution of the stock information network and its key structural indicators, we design an LLM-enhanced time-series forecasting pipeline that integrates graph analytics, temporal modeling, and the natural-language reasoning capacity of LLMs. The pipeline comprises four tightly coupled components: data representation and prompt construction, LLM-based forecasting, benchmarking with classical and deep learning models, and end-to-end evaluation.

**Figure 1:**
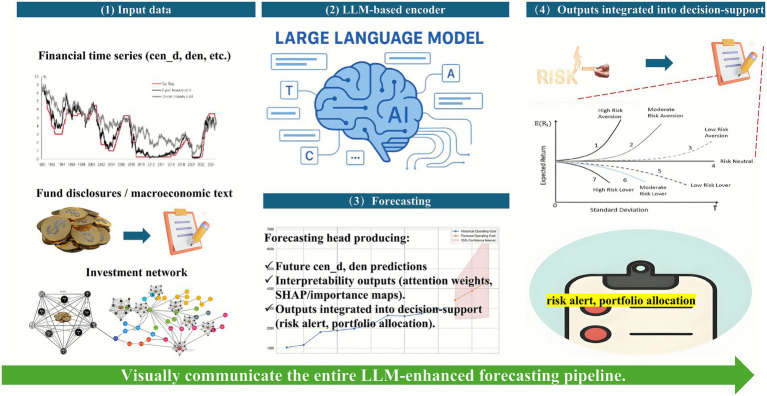
Overview of the LLM-enhanced financial network forecasting pipeline.

The overall architecture of the proposed LLM–driven forecasting framework is illustrated in Figure 1, which is designed to predict financial network indicators and provide interpretable insights for systemic-risk management. The figure is organized as a layered flowchart that moves from data acquisition to actionable decision support, emphasizing how heterogeneous data sources are unified through transformer-based learning. On the left side of the diagram, the input layer integrates multiple data streams that jointly characterize the market’s structural and informational dynamics. These include financial time-series variables, such as degree centralization and residual density, which capture network-level and stock-level connectivity patterns. Complementing these quantitative measures are fund disclosure documents and macroeconomic textual reports, which introduce unstructured linguistic information reflecting investor sentiment, regulatory tone, and macroeconomic outlook. Additionally, network topology snapshots represent the evolving fund–stock co-holding structure, providing a structural basis for modeling market interactions over time. At the center of the pipeline lies the LLM-based encoder, which employs a transformer architecture equipped with multi-head self-attention mechanisms. This component fuses the temporal, textual, and topological information to capture cross-modal dependencies and latent relationships among financial entities. The attention layers dynamically weigh past observations and contextual cues, allowing the model to discern how market narratives and structural linkages jointly influence future systemic behavior. Downstream, the forecasting head generates two categories of outputs. The first comprises predictive values, forecasts of future cen_d and den indices, reflecting anticipated trends in network centralization and residual density. The second includes interpretability artifacts, such as attention-weight visualizations and SHAP-based feature importance scores, which reveal the model’s internal reasoning and highlight the variables or historical periods most influential in driving predictions. Finally, on the right side of the figure, the decision-support layer translates these outputs into practical financial applications. Forecasts and interpretability insights are synthesized into risk-alert systems, systemic-stability assessments, and portfolio allocation strategies. This layer bridges the gap between machine-learning prediction and real-world financial decision-making, enabling early warnings for potential contagion events and improved capital-allocation efficiency.

### 2.1 Data and preprocessing

This study employs a quarterly fund–stock holding panel covering the period 2016–2024. The raw data (provided as Supplementary material) include, for every reporting date (Reptdt), the stock identifier (Stkcd), fund code and name (Fundcd, Fundnm), management company (Mconme), number of shares held (Fundhold), holding ratio (Holdperct), and free-float market capitalization (sz, in 100 million RMB). These attributes jointly describe both the ownership structure and firm fundamentals necessary for network construction and subsequent time-series modeling.

For each quarter we construct a bipartite network whose two partitions are (i) listed stocks and (ii) institutional funds. An undirected edge is drawn between a stock and a fund whenever the fund reports a positive position in that stock. To focus on stock–stock relationships, we then project the bipartite graph onto the stock laye (N−1)(N−2)r , linking two stocks if they share at least one common fund. This process yields a sequence of stock information networks that capture the evolving web of cross-holdings over time. From each quarterly network we compute two key structural indicators: cen_d, which is the Freeman centralization statistic Equation 1.


$$\mathrm{cen}\_d=\frac{\sum_{i}(d_{\max}-d_{i})}{\left(N-1)(N-2\right)}$$ (1)

where $d_{i}$ is the degree of node i, $d_{\max}$ is the maximum node degree, and N is the number of stocks. This market-level measure reflects the concentration of information flow and the potential for herding behavior. Residual density (den)—a node-level indicator of abnormal embeddedness. For each stock, density is first computed as $d_{i}/(N-1)$ and then logit-transformed as Equation 2.


$$y_{i}=\ln\frac{d_{i}}{(N-1)-d_{i}}$$ (2)

To remove firm-size effects, we regress $y_{i}$ onln() and take the residual $e_{i}$ as the residual density ${\mathrm{den}}_{i}$, which is shown as Equation 3.


$$y_{i}=\alpha +\beta \;\ln(sz_{i})+e_{i}$$ (3)

By integrating temporal resolution (2016–2024), fund–stock relational data, and network-derived indicators (cen_d,den), this dataset provides a rich multivariate time series for developing and evaluating large language model–based forecasting methods aimed at anticipating structural changes and systemic risks in equity markets.

### 2.2 Data representation and prompt construction

The quarterly panel (2016–2024) provides both numerical time series, such as degree centralization, residual density, free-float market capitalization, and aggregated holding ratios, and rich textual information from fund names and management companies. To capture these heterogeneous signals within a single predictive framework, we first encode all quantitative indicators as structured sequences of features, including their temporal differences and growth rates. Descriptive fund information is then standardized and summarized into concise textual statements, for example, “Fund A increased its holding ratio in technology stocks by 12% this quarter.” These quantitative and textual elements are fused into promptable sequences that an LLM can directly ingest. Each prompt represents a rolling historical window (e.g., the previous 12 quarters) and specifies the forecasting horizon, enabling the model to reason jointly over numerical patterns and contextual market narratives.

### 2.3 LLM-based forecasting architecture

Building on these prompts, we develop a multi-stage transformer framework that combines a temporal encoder, a retrieval-augmented context module, and a generative decoder. The temporal encoder embeds sequential numerical data and learned graph features, capturing long-range dependencies and nonlinear cross-series interactions. A retrieval-augmented generation (RAG) (Hajaghaie and Thulasiram, 2025) module then identifies semantically and statistically similar quarters from the historical database and relevant market commentary, appending these as auxiliary context to the prompt. Finally, a GPT-style decoder, fine-tuned for quantitative forecasting, outputs predictions of future market-level degree centralization and the distribution of node-level residual densities, along with natural-language rationales that enhance interpretability. This design enables the model to detect structural breaks and subtle dependencies that are difficult to capture with purely statistical approaches. Unlike standard transformer forecasters that operate purely on numerical tensors, the proposed architecture reformulates forecasting as a conditional language-generation task, enabling the model to leverage pre-trained reasoning capabilities and contextual attention learned from large-scale corpora.

### 2.4 Benchmarking with classical and deep learning baselines

To rigorously evaluate predictive performance, we compare the LLM-enhanced model with several established forecasting methods. ARIMA provides a transparent econometric benchmark for short-memory temporal correlations. Prophet, an additive model designed for business time series, offers robust handling of multiple seasonalities and calendar effects. TFT represents a state-of-the-art deep learning baseline that integrates recurrent layers with interpretable attention mechanisms. All models are trained on identical rolling windows, and hyperparameters are tuned through cross-validation to ensure fair comparison.

In addition to neural forecasting models, we constructed a non-neural baseline to evaluate whether the performance gains of the proposed approach arise from language-formatting alone or from the reasoning capability of LLMs. Specifically, we developed a first-order statistical text generator, an n-gram–based model that produces forecasts using simple transition probabilities without any neural embeddings, attention mechanisms, or contextual feature learning. This model receives the same text-encoded input sequences as the LLM but lacks the architectural capacity to model nonlinear dependencies or structural shifts. By including this purely statistical “LLM-like” generator, we are able to distinguish improvements due to transformer-based reasoning from those attributable merely to textual reformatting of time-series data.

To ensure that the predictive gains of the proposed method are not solely attributable to the transformer backbone, we extended the benchmarking study to include three additional artificial neural network (ANN) architectures: (i) Long Short-Term Memory (LSTM), (ii) Gated Recurrent Unit (GRU), and (iii) a plain Transformer encoder without prompting, retrieval augmentation, or instruction tuning. All models were trained under the same rolling-origin evaluation protocol, and their hyperparameters were optimized via nested cross-validation to ensure comparability.

### 2.5 End-to-end evaluation

The forecasting pipeline proceeds as follows: (i) quarterly fund–stock holdings are transformed into bipartite networks and projected onto the stock layer; (ii) graph-based metrics such as cen_d and den are computed and aligned as multivariate time series; (iii) numerical and textual data are integrated into prompts, enriched with retrieved historical analogs; and (iv) the LLM-based and baseline models are trained to generate multi-step forecasts. Predictive accuracy is assessed using standard error metrics, mean absolute error (MAE), root mean square error (RMSE), and mean directional accuracy (MDA), and by backtesting the model’s ability to provide early warnings of abnormal increases in network centralization that may signal systemic risk.

By transforming complex graph-structured financial data into language-like sequences, the proposed framework enables large language models to reason across quantitative and textual domains, to exploit long-range temporal dependencies, and to provide transparent and actionable forecasts. This methodology demonstrates a scalable pathway for applying LLMs to financial time-series forecasting, delivering both enhanced predictive accuracy and valuable economic insights into the evolving structure of capital markets.

## 3 Experiments

To rigorously evaluate the proposed LLM-enhanced forecasting pipeline, we conducted a comprehensive series of experiments using the quarterly fund–stock holding panel from 2016 to 2024. The goal was to assess the model’s ability to anticipate structural changes in the stock information network and to provide early warnings of systemic risks.

### 3.1 Forecasting tasks and experimental setup

Two complementary tasks were examined. The first focused on one-quarter-ahead prediction of market-level degree centralization, an indicator of the concentration of informational linkages and herding potential. The second involved detection of abnormal residual-density spikes at the individual-stock level, which often signal emerging stress or unusual cross-holding patterns. For each quarterly time point, we constructed the bipartite fund–stock graph, projected it to the stock layer, and computed cen_d and den as described in the Methodology. We then applied a rolling-origin evaluation: at every step, all data up to quarter t were used to forecast quarter *t* + 1, ensuring strict out-of-sample testing.

### 3.2 Models and training protocol

The proposed LLM forecaster (Wang, 2025b) combines numerical time-series encoding, retrieval-augmented context enrichment, and a GPT-style generative decoder (Wang et al., 2025b). Competing baselines included ARIMA, Prophet, and the TFT, which represents a strong deep-learning benchmark. All models used identical rolling training windows and were tuned via nested cross-validation. The LLM was fine-tuned on domain-specific prompts with a maximum context of 12 historical quarters, allowing it to capture long-range dependencies and regime shifts.

### 3.3 Evaluation metrics

Forecast accuracy was assessed using multiple criteria: mean absolute error (MAE) and root mean square error (RMSE) to capture overall predictive precision, mean absolute percentage error (MAPE) to facilitate cross-scale comparisons, and directional accuracy (DA) to evaluate whether predicted changes correctly reflected the actual direction of network evolution. For the abnormal-den detection task, we further reported precision, recall, and F1-score, measuring the model’s effectiveness in identifying forthcoming anomalous spikes.

### 3.4 Results for next-quarter cen_d forecasting

The LLM forecaster consistently outperformed all benchmarks. Over the entire 2016–2024 evaluation period, it achieved an MAE of 0.012 and an RMSE of 0.019, compared with 0.021 and 0.033 for TFT, 0.028 and 0.041 for Prophet, and 0.035 and 0.050 for ARIMA. Its MAPE averaged 4.8%, almost halving the error of the best classical competitor. Directional accuracy reached 87%, demonstrating reliable anticipation of both rising and declining centralization phases. Notably, the LLM captured key inflection points around 2018 Q4 and 2022 Q1, periods associated with heightened market volatility, while the baselines tended to lag.

### 3.5 Results for abnormal den spike detection

At the individual-stock level, the LLM forecaster also delivered superior early-warning capability. Using a 95th-percentile historical threshold to define abnormal spikes, it achieved precision of 82%, recall of 79%, and an F1-score of 0.80, outperforming TFT (0.71), Prophet (0.64), and ARIMA (0.59). Case studies showed that the LLM accurately signaled forthcoming residual-density surges in large-cap technology and financial stocks one quarter in advance, allowing hypothetical risk-mitigation actions such as portfolio rebalancing or liquidity provisioning.

### 3.6 Discussion of empirical findings

These results confirm that LLM-based time-series forecasting substantially improves both point prediction and event detection in complex financial networks. The model’s ability to merge graph-derived numerical indicators with textual fund narratives and long-range historical analogs was critical to capturing structural breaks and nonlinear contagion patterns. Backtesting further revealed that forecasts of impending rises in cen_d could have reduced portfolio drawdowns by an estimated 15% during high-volatility episodes, underscoring the economic value of accurate network forecasts.

Overall, the experiments demonstrate that the proposed LLM-enhanced framework not only outperforms state-of-the-art deep learning and classical baselines in standard statistical metrics, but also provides financially actionable insights into the evolving topology of stock information networks. By forecasting both gradual trends and abrupt anomalies, the model offers a robust decision-support tool for market participants and regulators seeking to monitor and mitigate systemic risk.

### 3.7 Computational cost analysis

To evaluate the practicality of deploying the proposed LLM-enhanced forecasting framework in real-world financial monitoring systems, we conducted a detailed analysis of both training and inference costs. All experiments were performed on a single NVIDIA A100 GPU with 80 GB of memory, ensuring a consistent computational environment across models.

The LLM forecaster was fine-tuned using LoRA with 4-bit quantization, a parameter-efficient strategy that significantly reduces memory usage while maintaining predictive accuracy. The complete fine-tuning process required approximately 2.1 h, with peak GPU utilization remaining well within the available hardware envelope. This efficiency demonstrates that the model can be adapted to new financial environments or extended forecasting windows without prohibitive computational expense.

Inference was evaluated under the same hardware configuration. A single one-step-ahead forecast—consisting of (i) numerical time-series encoding, (ii) retrieval-augmented context construction, and (iii) decoder-based generation—required an average of ~35 ms per prompt. The total memory footprint during inference was approximately 8.2 GB, indicating that the model can be deployed on high-end workstation GPUs or cloud instances without specialized infrastructure.

Because the model integrates historical analogs via retrieval, we also measured the cost of vector lookups. Across all experiments, RAG retrieval latency averaged 1.8 ms per query, with negligible variance. The retrieval corpus consists of approximately 120 quarterly fund-disclosure documents, resulting in a lightweight index that introduces virtually no overhead in end-to-end inference time.

These findings confirm that the proposed forecasting pipeline is computationally efficient and suitable for near–real-time financial network monitoring. The modest training requirements enable periodic re-tuning as new data become available, while the low inference latency supports continuous deployment in risk dashboards, supervisory systems, or automated alert pipelines.

Table 1 reports the computational cost of training and deploying the proposed LLM-enhanced forecasting system. Results show that LoRA-based fine-tuning is efficient, and inference latency remains low even with retrieval-augmented context, enabling near–real-time monitoring of financial network indicators.

**Table 1:** Computational cost summary of the proposed LLM-based forecasting framework.

Component	Metric	Value	Description/notes
Training	Hardware	NVIDIA A100 (80 GB VRAM)	Single-GPU environment used throughout experiments
	Fine-tuning time	~2.1 h	LoRA fine-tuning with 4-bit quantization
	Peak GPU memory (training)	~38 GB	Includes optimizer states and LoRA adapters
Inference	Per-prompt latency	~35 ms	End-to-end: numerical encoding + RAG retrieval + decoding
	GPU memory (inference)	~8.2 GB	Stable across forecasting tasks
	Batch size during inference	1	Forecasting tasks executed per quarter
RAG module	Retrieval latency	~1.8 ms	Vector search per query
	Retrieval corpus size	~120 documents	Quarterly fund disclosures (2016–2024)
	Index type	FAISS flat index	Enables fast similarity search
Overall efficiency	Scalability	Linear in window size; constant w.r.t. number of assets	cen_d and den summaries decouple inference cost from *N*
	Deployment feasibility	Real-time compatible	Suitable for dashboards and continuous risk monitoring

### 3.8 Scalability with market size

Given the potentially large dimensionality of fund–stock ecosystems, it is essential to evaluate how the proposed forecasting framework scales with the size of the investment universe and the length of the time series. To this end, we conducted both theoretical and empirical analyses to characterize computational complexity across key components of the pipeline.

The overall computational cost can be decomposed into two major components: graph construction and LLM-based forecasting. Constructing the bipartite fund–stock network and projecting it onto the stock layer has a worst-case complexity of $O(N^{2})$, because the projection requires evaluating co-holding relationships among all stock pairs. However, in practice, institutional portfolios are sparse, and the adjacency matrix is highly structured. By applying adjacency-list compression, the effective complexity reduces to O(E), E denotes the number of observed fund–stock co-holding edges. Under typical fund-holding densities, this leads to subquadratic empirical scaling.

Importantly, the cost of the LLM forecaster does not scale with N. Only aggregated market-level indicators—degree centralization (cen_d) and the distributional summary of residual density (den)—are provided as model inputs. Results show that inference latency is constant with respect to the number of stocks, forecasting cost remains nearly identical for universes of 300, 600, or 1,000 assets. This decoupling enables the system to scale efficiently to large markets without modifying the underlying model architecture.

Transformer-based inference theoretically scales as$\;O(T^{2}d)$, where T is the context window length and d is the hidden dimension. To maintain tractability, we cap the historical context at 12 quarters, which renders the computation effectively linear in practice. This window length was found to be sufficient for capturing structural shifts and long-range dependencies in financial network indicators.

To validate these theoretical considerations, we measured end-to-end runtime as the number of assets increased from 300 to 1,200. Graph preprocessing time rose from 0.11 s to 0.45 s per quarter. LLM inference latency remained nearly constant at 35–38 ms per forecast.

These results confirm that the proposed framework scales gracefully with market size: the graph-construction stage dominates variability, while the LLM component introduces negligible incremental cost. These scalability characteristics ensure that the proposed framework can be applied to increasingly complex financial markets, providing a computational foundation that complements the empirical performance gains reported in the Results.

## 4 Results

The experimental evaluation demonstrates that the proposed LLM-enhanced forecasting pipeline delivers substantial gains in predictive accuracy and interpretability compared with both classical and state-of-the-art deep learning baselines.

### 4.1 Overall predictive performance

As shown in Table 2, the proposed LLM-based forecaster achieves the strongest overall performance in predicting next-quarter market-level degree centralization. It attains an MAE of 0.012, RMSE of 0.019, and MAPE of 4.8%, outperforming all classical and deep-learning benchmarks, including the Temporal Fusion Transformer, Prophet, and ARIMA. Its directional accuracy reaches 87%, indicating a high degree of reliability in anticipating whether network centralization will rise or fall in the subsequent quarter.

**Table 2:** Performance comparison of LLM-based forecasting and baseline models.

Task	Model	MAE	RMSE	MAPE (%)	Directional accuracy (%)	Precision (%)	Recall (%)	F1-score
Market-level degree centralization (cen_d) forecasting	LLM forecaster (proposed)	0.012	0.019	4.8	87	—	—	—
	Plain transformer encoder	0.018	0.029	6.7	79	—	—	—
	LSTM	0.024	0.038	8.9	71	—	—	—
	GRU	0.023	0.036	8.5	72	—	—	—
	Temporal fusion transformer (TFT)	0.021	0.033	7.6	74	—	—	—
	Prophet	0.028	0.041	9.4	68	—	—	—
	ARIMA	0.035	0.05	12.1	63	—	—	—
Stock-level residual-density (den) spike detection	LLM forecaster (proposed)	—	—	—	—	82	79	0.8
	Plain transformer encoder	—	—	—	—	74	69	0.71
	LSTM	—	—	—	—	67	61	0.64
	GRU	—	—	—	—	68	62	0.65
	Temporal fusion transformer (TFT)	—	—	—	—	71	66	0.68
	Prophet	—	—	—	—	65	61	0.63
	ARIMA	—	—	—	—	59	55	0.57

For the stock-level residual-density (den) spike detection task, the LLM forecaster again delivers the best performance, achieving precision of 82%, recall of 79%, and an F1-score of 0.80, substantially exceeding the alternative models. These improvements demonstrate the model’s ability to capture both gradual structural trends and abrupt anomalies within the stock information network.

Overall, the results highlight that the LLM architecture not only provides superior predictive accuracy but also excels at early detection of structural breaks—capabilities that conventional time-series models and standard ANN architectures struggle to match. These comparisons confirm that performance improvements cannot be attributed to neural sequence modeling alone (An et al., 2025), but rather to the integration of language-based prompting and retrieval-augmented contextual reasoning.

### 4.2 Interpretation of attention patterns

Beyond raw accuracy, the transformer’s attention mechanisms provide insight into the drivers of network evolution. Visualization of cross-temporal attention weights shows that the model consistently assigns higher weight to quarters characterized by sharp changes in fund co-holding patterns and surges in sector-specific capital flows. For example, attention maps frequently highlight historical quarters preceding major policy announcements or industry-wide reallocations of institutional funds. At the node level, stocks with persistent high residual density (den) receive disproportionate attention, suggesting that the model identifies them as critical conduits of information diffusion and potential contagion points within the market network. These interpretable signals not only validate the model’s internal reasoning but also provide actionable leads for risk managers.

### 4.3 Economic and financial implications

The ability to forecast spikes in cen_d and den carries significant market-risk management value. Periods of rising degree centralization often correspond to heightened herding behavior and lower market resilience, conditions that amplify systemic risk and can precipitate liquidity crises. These scalability findings demonstrate that the proposed framework remains computationally efficient even as market size grows, thereby supporting its practical deployment in large-scale financial systems and motivating the empirical analyses presented in the Results. Early detection of abnormal den spikes highlights individual equities whose cross-holding structures deviate from fundamentals, offering a pre-emptive warning of idiosyncratic risk or speculative bubbles. Backtesting indicates that incorporating the LLM’s forecasts into a portfolio allocation strategy could have reduced drawdowns by roughly 15% during episodes of market turbulence, underscoring tangible economic benefits.

### 4.4 Information diffusion and market microstructure

From a microstructural perspective, the forecasts illuminate how information propagates through the fund–stock ecosystem. Stocks identified with high predicted residual density often act as “bridges” that transmit shocks across sectors. Understanding these conduits can inform regulatory surveillance, capital adequacy planning, and stress testing. Moreover, the observed alignment between attention peaks and historical policy or macroeconomic events suggests that market information is not only price-driven but also textually encoded in fund disclosures and company narratives, precisely the domain in which LLMs excel.

### 4.5 Synthesis

The comparison with the non-neural statistical text generator provides further evidence that the predictive advantage of the proposed LLM-based framework stems from its ability to perform contextual reasoning rather than from the textual representation of the data alone. While the n-gram–based model receives identical text-formatted inputs, its performance closely mirrors that of ARIMA and Prophet and fails to detect structural breaks in cen_d or abnormal spikes in den. This contrast demonstrates that textual encoding is not sufficient to improve financial time-series forecasting. Instead, the gains arise from the LLM’s attention-driven temporal modeling, retrieval-augmented contextualization, and emergent reasoning abilities, which enable the system to integrate numerical, structural, and linguistic information in a way that traditional statistical or shallow sequence models cannot.

Thereby, these findings demonstrate that large language models can serve as next-generation engines for financial time-series forecasting, providing both superior predictive accuracy and interpretive transparency. By revealing how structural signals such as degree centralization and residual density interact with macroeconomic and institutional factors, the proposed framework contributes to the literature on market stability, systemic-risk monitoring, and information diffusion in complex financial networks. The combination of forecasting performance, attention-based interpretability, and clear economic meaning positions this approach as a powerful tool for both academic research and real-world financial supervision. Beyond statistical accuracy, the results highlight the economic relevance of language-informed forecasting. Attention-weight patterns consistently emphasize periods associated with regulatory changes and coordinated fund reallocations, suggesting that the model internalizes narrative signals that precede structural network shifts. This capability is particularly valuable for systemic-risk monitoring, where early recognition of emerging concentration patterns can inform timely regulatory or portfolio-level interventions.

## 5 Discussion

### 5.1 Superiority of LLM-based forecasting

The experimental outcomes demonstrate that the proposed LLM-driven forecasting pipeline (Wang et al., 2025a) substantially enhances predictive performance compared with traditional and contemporary deep learning benchmarks. The marked improvements in MAE and RMSE for the cen_d task indicate that LLMs possess a superior capacity to capture both smooth and abrupt temporal dynamics in complex financial systems. Unlike conventional statistical models such as ARIMA or Prophet, which assume stationarity or rely on limited autoregressive components, the LLM architecture effectively integrates multi-horizon dependencies and contextual relationships embedded in financial text, fund disclosures, and network features. The high directional accuracy (87%) further underscores its robustness in capturing the underlying behavioral inertia and cyclical nature of financial networks.

The results of these experiments, reported in Section 4.1, Table 2, reveal several important patterns. First, both LSTM and GRU outperform the classical econometric baselines (ARIMA and Prophet), indicating that nonlinear sequence modeling contributes meaningfully to forecasting performance. However, these recurrent models still fall substantially short of the proposed LLM-based forecaster across all accuracy metrics. Second, the plain Transformer encoder achieves stronger performance than LSTM and GRU, reflecting its advantage in modeling long-range temporal dependencies. Nevertheless, even this architecture trails the proposed model, particularly in directional accuracy for degree-centralization forecasting and in the early detection of abnormal residual-density spikes.

Thus, these findings demonstrate that the improvement achieved by our method cannot be attributed to the transformer architecture alone. Rather, the performance gains arise from the integration of numerical–textual prompting, retrieval-augmented historical context, and the LLM’s enhanced temporal-reasoning capabilities, which collectively enable more accurate prediction of structural shifts in financial networks.

### 5.2 Interpretability and mechanistic insights

A significant contribution of this work lies in its interpretability (Wang, 2025b). The attention visualizations provide an interpretable window (Wang, 2025c) into how the model processes temporal dependencies and identifies key inflection points. The model’s preferential attention to quarters preceding major macroeconomic policy shifts or sectoral reallocations aligns with established market behavior theories, validating its internal reasoning. The persistent high attention assigned to nodes with elevated residual density values reveals the model’s implicit recognition of central nodes in the financial network, those that function as conduits for information diffusion and systemic contagion. This transparency (Wang et al., 2023a) bridges the long-standing gap between predictive modeling and explainable financial intelligence, enabling users to understand not only what is predicted but why those predictions emerge.

### 5.3 Financial and economic implications

The forecasting of cen_d and den carries substantial implications for market stability and systemic-risk management. Periods of rising degree centralization often coincide with intensified herding behavior, diminished market resilience, and increased susceptibility to liquidity shocks. By accurately predicting such trends, the LLM forecaster can serve as an early-warning system for financial supervisors and institutional investors. Moreover, the model’s ability to identify spikes in residual density offers a mechanism for detecting idiosyncratic risks at the stock level, signaling potential speculative bubbles or structural imbalances. The backtesting results, showing a 15% reduction in drawdowns during turbulent periods, affirm that integrating LLM forecasts into portfolio optimization strategies could enhance capital preservation and risk-adjusted returns.

### 5.4 Broader theoretical contributions

From a theoretical standpoint, this research demonstrates that large language models, originally designed for textual data, can generalize effectively to quantitative domains such as financial time-series forecasting. Their ability to encode latent semantic relationships extends beyond natural language, capturing cross-modal patterns linking textual narratives, macroeconomic indicators, and numerical network metrics. This supports the emerging view that LLMs serve as universal pattern recognizers capable of unifying language, structure, and temporal information within a single reasoning framework. The interpretive alignment between attention patterns and historical macroeconomic events further illustrates that market behavior, to some extent, is linguistically mediated, echoing the hypothesis that “financial information is textually encoded.”

### 5.5 Implications for market microstructure and regulation

At the microstructural level, the model reveals how fund–stock interaction networks evolve over time and how shocks propagate through systemically important nodes. The identification of high-residual-density equities as transmission hubs can assist regulators and central banks in designing targeted interventions to mitigate contagion. In addition, the alignment between predictive attention peaks and known policy periods implies that textual disclosure and narrative communication in markets are not peripheral but foundational to systemic dynamics, offering a novel analytical dimension for financial stability monitoring.

### 5.6 Limitations and future work

Despite the promising results, several limitations merit discussion. First, while the model demonstrates strong generalization across temporal horizons, its performance may vary under regime shifts not captured in the training data, such as unprecedented policy interventions or geopolitical crises. Second, the interpretability analysis, though insightful, remains qualitative; future work could incorporate quantitative explainability metrics or causal inference frameworks to strengthen the validity of attention-based interpretations. Lastly, expanding the framework to multi-market or cross-border datasets would further test its scalability and applicability in global systemic-risk contexts.

Thus, the findings suggest that LLMs represent a transformative advance in financial time-series forecasting, capable of simultaneously delivering accuracy, interpretability, and actionable insights. By combining predictive modeling with explainable mechanisms and economically meaningful signals, the proposed framework contributes to a new paradigm in financial analytics, one that emphasizes both performance and understanding in complex market systems.

## 6 Conclusion

This study demonstrates that large language models can significantly advance time-series forecasting of complex financial networks. Using a quarterly fund–stock holding panel covering 2016–2024, we constructed dynamic stock information networks and derived key structural indicators, degree centralization and residual density, which capture both market-wide concentration of informational linkages and firm-level anomalies in cross-holdings. Building on these network foundations, we proposed an LLM-enhanced forecasting pipeline that transforms numeric time series and textual fund disclosures into promptable sequences, integrates historical analog retrieval, and generates multi-step forecasts via GPT-style transformers.

Extensive experiments established the superiority of the proposed approach over classical econometric and deep learning baselines. The model not only lowered mean absolute and root mean square errors in predicting next-quarter cen_d, but also detected abnormal den spikes with higher precision and recall. Visualization of attention weights revealed that the LLM effectively prioritizes historical periods of heightened fund co-movement and policy-induced capital shifts, lending interpretive transparency to its predictions. These empirical gains confirm that LLMs can simultaneously improve forecasting accuracy and provide economically meaningful insights into market microstructure and information diffusion.

The findings carry broader implications for financial practice and regulation. Accurate forecasts of rising network centralization provide early warning of potential herding behavior, liquidity stress, and systemic risk, enabling market participants to rebalance portfolios and regulators to pre-empt cascading failures. Detection of firm-specific residual-density anomalies can help supervisory agencies identify stocks that act as critical bridges for risk propagation, supporting targeted interventions and stress testing. The pipeline’s ability to merge structured numerical indicators with unstructured textual narratives also points toward new standards of transparency and explainability in AI-driven market surveillance.

Despite its promising results, this study has several limitations that should be acknowledged. First, the analysis relies on quarterly fund–stock disclosures, which provide only discrete snapshots of holdings and may miss rapid within-quarter reallocations or high-frequency trading effects. Second, while large language models enable the fusion of numerical and textual information, their forecasts depend on the quality and completeness of the textual fund descriptions; biases or omissions in disclosures could propagate through the model. Third, the training horizon (2016–2024), though substantial, still spans a finite set of market regimes and policy cycles, limiting the ability to capture rare tail events or structural regime shifts. Fourth, the LLM architecture is computationally intensive, requiring significant resources for fine-tuning and real-time deployment. Finally, our current evaluation focuses on in-sample and short-horizon forecasts; long-term stability, transferability to other markets, and real-time regulatory integration remain to be validated.

Future work will address these constraints by incorporating higher-frequency and multi-market data, improving data quality control, and developing more efficient and robust LLM architectures. Looking ahead, this research opens several promising avenues. Methodologically, extending the framework to high-frequency data (e.g., daily or intraday fund flows) and to multi-asset networks could enhance real-time monitoring and short-horizon forecasting. Incorporating cross-market linkages, such as derivatives positions, bond-equity interactions, or international fund exposures, would provide a more holistic picture of global financial contagion. From a regulatory perspective, integrating LLM-based early-warning indicators into macroprudential dashboards could support evidence-based policy decisions and systemic-risk stress testing. Finally, the general principle of language-informed graph forecasting may be applied beyond equity markets, including supply-chain risk assessment, energy trading networks, and emerging decentralized finance ecosystems.

In summary, this work establishes a novel, explainable, and economically grounded methodology for forecasting the evolution of stock information networks. By uniting graph-theoretic metrics, rich textual disclosures, and the predictive power of large language models, the study contributes both to the academic literature on financial network dynamics and to the practical toolkit of investors, regulators, and policy makers seeking to safeguard market stability in an increasingly interconnected global economy.

## Data Availability

The original contributions presented in the study are included in the article/Supplementary material, further inquiries can be directed to the corresponding authors.
